# The (a)typical burden of COVID-19 pandemic scenario in Autism Spectrum Disorder

**DOI:** 10.1038/s41598-021-01907-x

**Published:** 2021-11-22

**Authors:** Lucia Fumagalli, Monica Nicoli, Laura Villa, Valentina Riva, Michele Vicovaro, Luca Casartelli

**Affiliations:** 1 Theoretical and Cognitive Neuroscience Unit-Child Psychopathology Department, Scientific Institute, IRCCS E. Medea, Bosisio Parini, Italy; 2 Child Psychopathology Unit, Scientific Institute, IRCCS E. Medea, Bosisio Parini, Italy; 3 Department of General Psychology, University of Padova, Padova, Italy

**Keywords:** Psychology, Health care

## Abstract

Psychological and mental health consequences of large-scale anti-contagion policies are assuming strong relevance in the COVID-19 pandemic. We proposed a specific focus on a large sample of children with Autism Spectrum Disorder (ASD), developing an ad hoc instrument to investigate *changes* occurred in specific *(sub-)domains* during a period of national lockdown (Italy). Our questionnaire, named *AutiStress,* is both *context-specific* (being set in the COVID-19 pandemic scenario) and *condition-specific* (being structured taking into account the *autistic functioning* peculiarities in the paediatric age). An age- and gender-matched group of neurotypical (TD) controls was also provided. As expected, the severe lockdown policies had a general negative impact both on ASD and TD children, reflecting the obvious burden of the pandemic situation. However, our findings also indicate that children with ASD experienced more *positive* changes than TD ones. Noteworthy, we report a thought-provoking double dissociation in the context-specific predictor (i.e., accessibility to private outdoor spaces), indicating that it impacts differently on the two groups. Focusing on the ASD group, results suggest a condition-specific impact of the COVID-19 pandemic on *core* autistic *(sub-)domains*. Taken together, our data call for a multi-layered, context- and condition-specific analysis of the pandemic burden beyond any oversimplification.

## Introduction

No doubt that the health, social and economic cost of the COVID-19 pandemic has had a worldwide impact on our lives, and it deeply set the course of biomedical research in 2020 providing extraordinary rapid insights on the severe acute respiratory syndrome coronavirus 2 (SARS-CoV-2) biology, diagnostics, and treatment^1–3^. Among the most insidious indirect effects of this pandemic, there are psychological and mental health consequences of large-scale anti-contagion policies^4–6^.

Epidemic modelling on virus spread is crucial to balance the significant beneficial health outcomes of extensive anti-contagion policies in slowing the growth rate of infections and their unavoidable psycho-socio-economic cost^7,8^. Although it is ideally clear that decision-makers should balance health benefits of these policies and psycho-socio-economic consequences of restrictions, how this should be practically provided is extremely challenging and highly debated^9–11^. This balance is even more complex to achieve when we deal with vulnerable populations such as, for example, individuals with previous history of neuropsychiatric conditions that for multifaceted factors may be potentially more affected by the pandemic^12^. Here, we propose a specific focus on a well-characterized sample of children with Autism Spectrum Disorder (ASD) living in the Northern Italy (Lombardy region), that has been one of the first and major pandemic epicenters in Europe^13^. This will provide not only additional insights on this extremely complex and multi-layered balance, but it offers the unique possibility to explore the impact of unprecedented restriction measures on children with ASD using a clinically driven approach based on peculiarities of the autistic functioning.

Following the initial mild restrictions circumscribed to specific areas (notably the Lombardy region), in 2 weeks (8th–22nd March, 2020) the Italian government implemented a series of more and more severe public-health measures. These anti-contagion policies took shape a scenario of initially local and then national lockdown^8,14^. Since May 2020, easing restrictions have been gradually provided allowing for example outdoor workout and the reopening of commercial activities. This scenario circumscribes the so-called COVID-19 first wave, and we limit our analysis to this specific period. Italian anti-contagion policies have been considered among the most stringent by the Oxford COVID-19 Government Response Tracker (OxCGRT), an index collecting information on country-specific governmental responses^11^.

ASD is an early emerging neurodevelopmental condition characterized by heterogenous phenotypical manifestations in the social domain, restrict and repetitive patterns of behaviours, and peculiarities in the sensory processing^15,16^. Beyond this largely shared agreement about the generic clinical features, both the pathophysiology and subtler phenotypical aspects of ASD are far to be fully clarified. Noteworthy, recent clinical and experimental approaches raised the hypothesis that inflexibility of learning/thinking/acting may represent a key component in explaining the heterogenous range of autistic behavioural and neurocognitive manifestations^17–20^. This phenomenology of the autistic phenotypes helps to overcome the simplistic view of ASD as a condition *primarily* characterized by social difficulties. In turn, it suggests that the constellation of behavioural “social” symptoms may represent an epiphenomenon of more radical peculiarities in dealing with the social dynamics, potentially reflecting (or reflected by) atypicalities in sensory, perceptual, and motor functioning^21–23^. Most refined clinical approaches on ASD seem to imply that the *autistic functioning* (i.e., the ASD individuals’ peculiar way of learning/thinking/acting) should be considered to evaluate the impact of potentially stressful situations. Thus, both the unprecedented pandemic COVID-19 scenario and the underestimated peculiarities of the autistic functioning call for a tailored approach in investigating the consequences of severe lockdown policies in our children with ASD sample. For these reasons, we provided an ad hoc questionnaire, named *AutiStress*, to explore the pandemic impact in a spectrum of *domains*, each characterized by specific *sub-domains* ultimately referring to distinct *types-of-change* (Table S1). Notably, this framework differentiates *core* Vs. *non-core* autistic features that basically reflect *domains* or *sub-domains* specifically/non-specifically ascribable to the autistic functioning. This aimed to go beyond the largely foreseeable and simplistic conclusions about a generic negative impact of the pandemic. To do it, we tried to control as well as possible the assessment, focusing on a well-characterized sample of children with ASD, with a relatively limited range of age, living in a well-defined geographic area in which the anti-contagion policies applied by the government were identical and the perceived general pandemic burden comparable. In addition, all children with ASD received readapted and personalized remote clinical support during the pandemic by our clinical staff. To minimize confound effects, we limit data collection to an initial tight window in which the anti-contagion policies were most severe (3rd–29th April, 2020). Noteworthy, we considered not only usual variables (e.g., age, cognitive level) but also particular ones taking into account the contextual *stay-at-home* scenario (e.g., access to private outdoor space). A group of neurotypical children was also provided as control group.

A priori, negative effects of the lockdown policies on our pediatric ASD sample were surely expected, in agreement with seminal perspective views^24^, and also in line with first empirical reports in the literature^25–28^. One may hypothesize that the strict adherence to routines frequently reported in children with ASD may be affected by sudden restrictions, and these may also prevent the access to usual reinforcers (e.g., swimming, going to the playground) with cascade negative effects on children with ASD’s compliance in daily-life activities or homework. However, and going beyond a simplistic and monolithic approach to this complex scenario, children may also benefit from limited contextual/external stimulations. *AutiStress* tries to tackle this complex and multi-layered scenario without neglecting the peculiarities of the autistic functioning, aiming to provide useful insights to manage the current pandemic, and also unwanted (but unfortunately not unrealistic) future emergency situations.

## Methods

### Participants

The final sample included 178 children with ASD (mean age ± SD = 5.44 ± 1.81 years, age range 2–9 years, males = 146) and 86 typically developing (TD) children (mean age ± SD = 5.00 ± 1.56 years, age range 2–9 years, males = 63). No differences between the ASD and the age- and gender-matched TD group were found on demographic variables (age: p = 0.06; gender: p = 0.10). Please note that the initial sample consisted of 178 children with ASD and 150 TD children (mean age ± SD = 3.92 ± 1.92 years, males = 81). Due to significant inter-group differences in age and gender variables (both ps < 0.001), we age- and gender-matched the two samples. Being the effects of severe anti-contagion policies on the ASD group our main focus, we chose to keep constant the ASD sample to maximise statistical power in ASD intra-group analyses. All children with ASD were in charge at the Scientific Institute IRCCS Medea (Bosisio Parini, Lecco; Italy) following distinct clinical protocols. Noteworthy, all children included in this sample continued to receive readapted and personalized remote clinical support by our clinical staff also during the most severe pandemic phases. IRCCS Medea is a renowned paediatric research hospital specialized in neurological, neuropsychiatric and neurodevelopmental disorders. Child Psychopathology Unit at the IRCCS Medea represents a unique and privileged observatory for ASD in Italy. It is recognized as “Pivot Center” for early detection and intervention in the Lombardy Region, and it has specific clinical departments using gold-standard clinical procedures for diagnosis (> 250 new ASD diagnosis per year) and behavioural evidence-based protocols for rehabilitation (e.g., NOAH project, New Organization for Autism Healthcare: ≈ 280 children with ASD between 2–6 years old enrolled in a 3-year program). In sum, our inclusion criterion for the ASD sample was the fact to be in charge at the IRCCS Medea in one of the clinical rehabilitative protocols for ASD (being formal diagnosis of ASD the prerequisite for accessing to these protocols). Concerning the TD group, we collected data in an anonymous way, and parents were requested to declare no prior history of psychiatric or neurological disorders in their children. We excluded any subjects for which parents declared any neurological or psychiatric diagnosis.

The study was approved by the IRCCS E.Medea-Ethical Committee, and it was conformed to the principles elucidated in the Declaration of Helsinki. Parents/legal guardians of all patients signed informed consent.

### *AutiStress* questionnaire: structure and coding

*AutiStress* questionnaire is an ad hoc created parent-report instrument that we originally developed and distributed in Italian (in the interest of international readers, we also provide an English version of the questionnaire in Table S1. Cfr. Supplementary Materials for details). *AutiStress* was developed to investigate *changes* occurred in specific *domains* and *sub-domains* (also defined as *types-of-change*) following the severe anti-contagion movement restrictions policies. It is *context-specific* being set in the COVID-19 pandemic scenario, and *condition-specific* being structured taking into account the autistic functioning peculiarities in the paediatric age. Thus, we differentiated core Vs. non-core autistic features (both in *domains* and in *sub-domains*) according to the idea that ASD is not a condition primarily characterized by phenotypical “social” manifestations. *AutiStress* was distributed using Google Forms between 3rd and 29th April, 2020.

After a preliminary description of the aims of the study, we collected general basic socio-demographic information. Notably, we asked about the house characteristics considering that accessibility to outdoor spaces may be a critical factor during a period characterized by severe movement restrictions. To maximize statistical power and to avoid misattributions due to ambiguous situations, we merged the four choices using a handy dichotomization between apartment regardless of the presence of the balcony (hereafter, apartment) and home with access to private outdoor spaces (hereafter, private garden).

We structured our requests into seven *domains* referring both to core autistic features (*Sensory Interests*; *Repetitive Behaviours*) and non-core ones (*Mood*; *Play*; *Eating Behaviour*; *Circadian Rhythm Sleep*; *Bowel and Bladder Control*). For each *domain*, parents were asked to report—if any—children’s behavioural changes as compared to pre-pandemic situation (e.g., “Did your child show any change in his/her mood? [yes/no]”). Following a negative answer, the online form moved to the subsequent *domain*. On the contrary, if parents replied positively in that *domain* they were asked to rate on a 5-point Likert scale how frequently several *domain*-related *types-of-change* occurred. Being a questionnaire that synergistically combined the efforts of clinicians engaged in remote support protocols and researchers, certain *AutiStress* requests were primarily driven by clinical purposes (notably to provide prompt and comprehensive monitoring for potential behavioural changes, even in less expected spheres). This was the case for example of the *Bowel and Bladder Control domain*, that was not extensively explored in our analyses due to very limited frequencies (12% in ASD group, 3% in TD group). Similarly, we simplified the *Sensory Interests domain* focusing exclusively on the main question (related to presence or absence of any change concerning sensory interests), without furtherly analysing *sub-domains* (i.e., each sense).

### Statistical analysis

Given the different nature of dependent variables between *domains* (dichotomous) and *sub-domains*/*types-of-change* (Likert scale), we tested our predictors of interest with different statistical models in Jamovi.

#### Domains

Generalized linear models were implemented to test main and interaction effects^29^. *Group* (ASD Vs. TD) and *House-Characteristics* (apartment Vs. private garden) entered in the model as dichotomous predictors, and *Age* as continuous covariate. The Group*House-Characteristics interaction also entered in the model. Moreover, for each sample we tested the effects of House-Characteristics and Age, in order to further assess the specific impact of these predictors within-group. In addition, being available children with ASD’s cognitive functioning measures from pre-pandemic clinical records, in the ASD group we ran the analysis also considering the *Cognitive Level* as a covariate. This predictor was dichotomized into ‘intellectual-disability’ Vs. ‘no-intellectual-disability’, to overcome variability in instruments used to measure cognitive functioning.

#### Sub-domains (types-of-change)

Analyses on the *sub-domains* were performed only on the residual sample of children reporting changes in the correspondent *domain*. Multiple linear regression models were implemented to test potential change in each *sub-domain*. Except for the Group*House-Characteristics interaction, which was not included here due to the small number of responses for some combinations of the levels of the two factors, for the analysis of the *sub-domains* we used the same predictors set that we used for the analysis of the *domains*. If the assumption of normality (K-S test) of the residuals was violated, then we dichotomized the continuous dependent variable into presence (i.e., from “1” to “4”) or absence (i.e., “0”) of change. Being *sub-domains* generally characterized by low frequencies in the right side of the Likert scale (i.e., responses “3–4”), we collapsed responses from “1” to “4”. Then a generalized linear model was used. We anticipate that, due to their peculiar nature, the *Mood sub-domains* were analysed somewhat differently from the other *sub-domains* (“Changes in non-core autistic features” section).

As we observed missing values, we also tested the hypothesis that missing data were randomly distributed (Missing Completely At Random–MCAR Analysis). If missing values were detected within at least one *sub-domain*, we ran the MCAR analysis within the correspondent *domain*. Control analyses confirmed that data are missing completely at random (*Circadian Rhythm Sleep*: p = 0.09; *Eating Behaviour*: p = 0.15; *Play*: p = 0.14; *Mood*: p = 0.62). No missing values were found in *Repetitive Behaviour domain*.

## Results

Results are presented according to the condition-specific and context-specific hallmarks of *AutiStress.* Thus, we focus on the core Vs. non-core autistic features distinction, making reference both to specific core autistic *domains* and *sub-domains*. In addition, we focus on the House-Characteristics predictor to consider peculiarities of the COVID-19 pandemic scenario. Nevertheless, detailed significant results that do not fall into this conceptual and clinically-driven framework are reported in Tables S2, S3, S4.

### Changes in non-core autistic features

**Figure 1:**
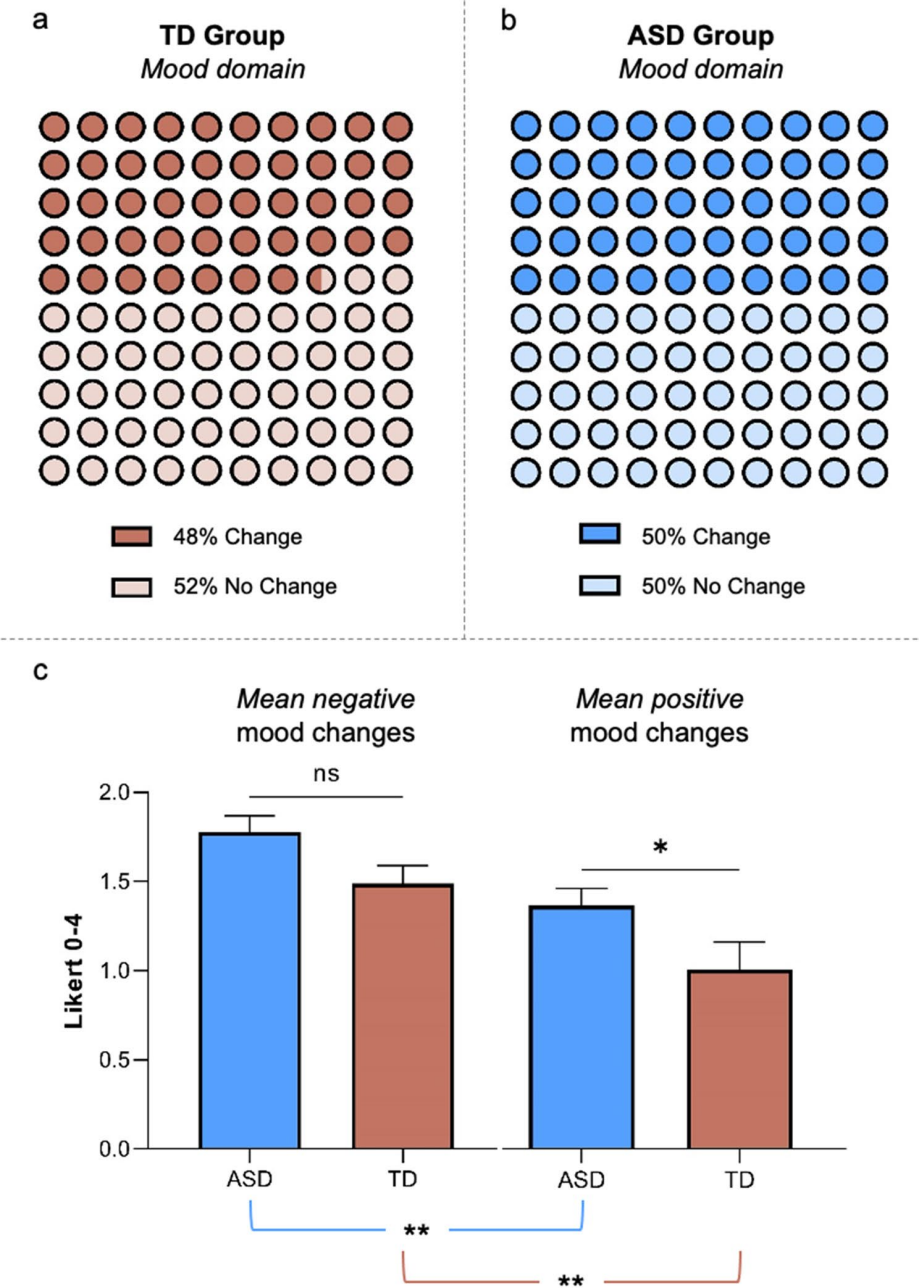
(**a**) Graphs illustrating TD children (48% [41/86]) that are reported to show changes in the *Mood domain*. (**b**) Graphs illustrating ASD children (50% [88/176]) that are reported to show changes in the *Mood domain*. (**c**) Graphs representing intra-group and inter-group comparisons in the *mean negative* and in the *mean positive* mood changes. Intra-group comparisons show that both ASD and TD children reported more *mean negative* than *mean positive* changes in mood (ASD group: p = 0.005; TD group: p = 0.004). Interestingly, there is no inter-group difference in the *mean negative* mood changes (ASD group: 1.78 ± 0.87; TD group: 1.49 ± 0.66; p = 0.10), but children with ASD reported more *positive* mood changes than TD children (ASD group: 1.31 ± 0.86; TD group: 0.97 ± 0.96; p = 0.019).*Statistical significance, p < 0.05; **statistical significance, p < 0.01; ns = no statistical significance. Bars represent standard errors of the mean (SEM).

*Mood domain* represents an unspecific and non-core autistic feature that may reliably help in characterizing the burden of the pandemic scenario both for ASD and TD children. We did not find Group difference in reporting changes in this *domain* (ASD: 50%[88/176]; TD: 48%[41/86]; LRT-χ^2^ = 0.08, p = 0.78) (Fig. 1a,b), nor any other significant effect of House-Characteristics (LRT-χ^2^ = 0.10, p = 0.75), Age (LRT-χ^2^ = 1.86, p = 0.17) or Group*House-Characteristics (LRT-χ^2^ = 0.62, p = 0.43). Concerning the within-group analyses, no significant effects of any predictor were found neither in the ASD nor in the TD group (ASD group: House-Characteristics LRT-χ^2^ = 0.75, p = 0.39; Age LRT-χ^2^ = 0.51, p = 0.48; Cognitive Level LRT-χ^2^ = 0.12, p = 0.73; TD group: House-Characteristics LRT-χ^2^ = 0.05, p = 0.83; Age LRT-χ^2^ = 1.86, p = 0.17).

Considering their peculiar nature, the *Mood sub-domains* were analysed differently from the other *sub-domains*. Specifically, instead of analysing separately the *types-of-change*, we first grouped them based on their *positive* Vs. *negative* value (Table S1). In other words, we averaged all the intra-subjects *negative* and all the intra-subjects *positive* mood changes. This allowed us to obtain valuable general information about *positive* and *negative* mood changes, independently of the specific nature of these changes. Because neither *mean positive* nor *mean negative* mood changes were normally distributed (ps < 0.01), we used Wilcoxon Paired Tests to compare ranks of *mean positive* Vs. *mean negative* differences in mood changes within each group. The results showed that both ASD and TD children reported more *mean negative* than *mean positive* changes in mood (ASD[N = 84]: 1.79 ± 0.86 > 1.30 ± 0.87, W = 2213, p = 0.005; TD[N = 40]: 1.50 ± 0.67 > 0.97 ± 0.96, W = 595, p = 0.004) (Fig. 1c).

In order to test the possible effect of the Group predictor on *mean negative* and *mean positive* mood changes, we used two separate Mann Whitney U-tests. If the U-test indicated a statistically significant effect of Group on the *mean positive* (*mean negative*) change, then we conducted a regression analysis with predictors Group, House-Characteristics, and Age on each of the *positive* (*negative*) *type-of-change*. Specifically, we used a generalized linear model if the assumption of normality of the residuals was violated and a simple linear model otherwise. In other words, analyses on the *positive* (*negative*) *types-of-change* were performed only if the Group predictor had a statistically significant effect on *mean positive* (*mean negative*) mood changes. In line with this post-hoc logic, we adjusted the significance threshold according to Bonferroni correction (*Positive* mood changes significant threshold: p = 0.05/3 = 0.017; *negative* mood changes significant threshold: p = 0.05/6 = 0.008). Note that, although these post-hoc analyses were focused on the possible effects of the Group predictor on each *type*-*of*-*change*, we also entered House-Characteristics and Age in the models in order to control for possible spurious correlation effects.

No effect of the Group predictor (ASD Vs. TD) emerged in the *mean negative* mood changes (ASD[N = 86]: 1.78 ± 0.87; TD[N = 41]: 1.49 ± 0.66; U = 1446, p = 0.10). In contrast, a distinct pattern between the ASD and TD group was found in the *mean positive mood* changes, with ASD children reporting more *positive* changes than TD ones (ASD[N = 86]: 1.31 ± 0.86; TD[N = 40]: 0.97 ± 0.96; U = 1279, p = 0.019) (Fig. 1c). In particular, post-hoc analysis on *positive types-of-change* revealed that ASD children were reported to show more changes than TD children in the *more calm* (ASD[N = 84]: 1.32 ± 1.02; TD[N = 39]: 0.69 ± 0.86; β = 0.76; p < 0.001), in the *more helpful* (ASD: 81%[67/83]; TD: 56%[22/39]; LRT-χ^2^ = 13.42, p < 0.001) and in the *more cooperating behaviours* (ASD: 73%[62/85]; TD: 63%[25/40]; LRT-χ^2^ = 4.57, p = 0.032) *sub-domains*.

**Figure 2:**
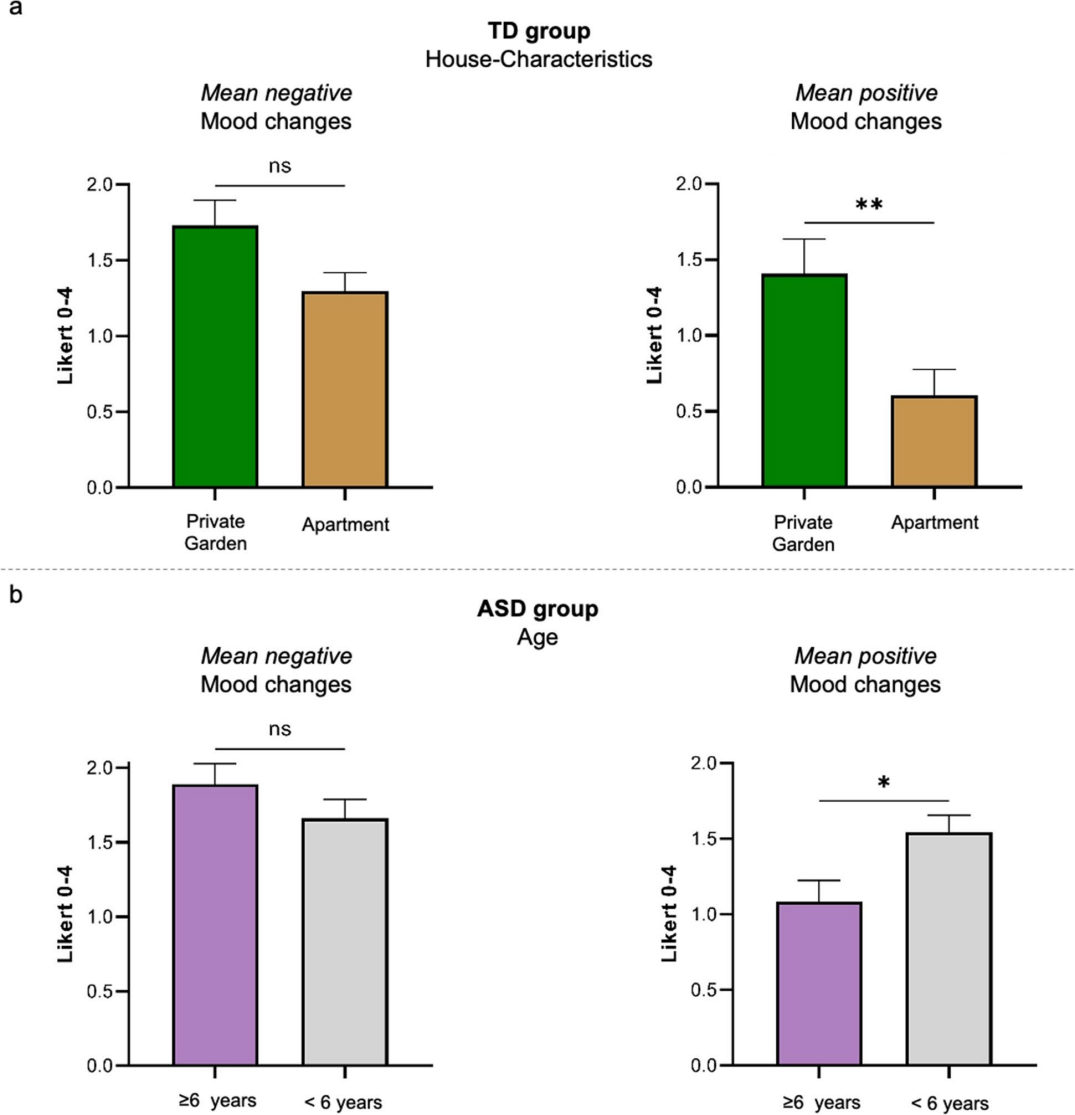
(**a**) Graphs illustrating the effects of House-Characteristics (private garden Vs. apartment) on the *mean negative* and *mean positive* mood changes within the TD group (p = 0.008). (**b**) Graphs illustrating the effects of Age (‘ ≥ 6 y.o.’ Vs. ‘ < 6 y.o.’) on the *mean negative* and the *mean positive* mood changes within the ASD group (p = 0.024). *Statistical significance, p < 0.05; **statistical significance, p < 0.01; ns = no statistical significance. Bars represent standard errors of the mean (SEM).

The possible effects of the House-Characteristics predictor (private garden Vs. apartment) and of the Age predictor on the *mean positive* and *mean negative* mood changes were separately analysed using the same logic as for the Group predictor. The effects of both predictors were tested separately on the TD and ASD group. Consistently with the choice of using the Mann Whitney U-test, the Age predictor was dichotomized (i.e., children ‘ < 6 y.o.’ and ‘ ≥ 6 y.o.’). As for the House-Characteristics factor, in the TD group we found a significant effect in the *mean positive* mood changes (private garden[N = 18]: 1.41 ± 0.97, apartment[N = 22]: 0.61 ± 0.80; U = 103, p = 0.008), and no significant effect in the *mean negative* mood changes (private garden[N = 18]: 1.73 ± 0.70, apartment[N = 23]: 1.30 ± 0.58; U = 138, p = 0.068) (Fig. 2a). Post-hoc analysis on *positive types-of-change*, with predictors House-Characteristics and Age, revealed that TD children having access to private outdoor places were reported to show more changes in the *more calm* (private garden: 71%[12/17]; apartment: 32%[7/22]; LRT-χ^2^ = 5.83, p = 0.016) and in the *more cooperating behaviours* (private garden: 89%[16/18]; apartment 41%[9/22]; LRT-χ^2^ = 10.60, p = 0.001) *sub-domains*. Also the *more helpful sub-domain* showed a comparable trend (private garden: 76%[13/17]; apartment: 41%[9/22]; LRT-χ^2^ = 5.04, p = 0.025), but it did not survive to the multiple comparison threshold (fixed at p = 0.017). No House-Characteristics significant effects were reported in the ASD group, neither on *mean positive* (private garden[N = 27]: 1.43 ± 0.97; apartment[N = 59]: 1.26 ± 0.81; U = 710, p = 0.42) nor on *mean negative* mood changes (private garden[N = 26]: 1.96 ± 0.86; apartment[N = 60]: 1.69 ± 0.87; U = 634, p = 0.17).

Concerning the Age predictor (‘ < 6 y.o.’ Vs. ‘ ≥ 6 y.o.’), in the ASD group we found a significant effect in the *mean positive* mood changes (‘ < 6 y.o.’[N = 43]: 1.54 ± 0.75; ‘ ≥ 6 y.o.’[N = 43]: 1.08 ± 0.92; U = 666, p = 0.024), and no significant effect in the *mean negative* mood changes (‘ < 6 y.o.’[N = 43]: 1.66 ± 0.82; ‘ ≥ 6 y.o.’[N = 43]: 1.89 ± 0.91; U = 789, p = 0.24) (Fig. 2b). In particular, regression analysis on the *positive types-of-change* with predictors Age (continuous), House-Characteristics, and Cognitive Level revealed that changes in the *more helpful* (β = -0.37; p < 0.001), in the *more calm* (β = -0.26; p = 0.015), and in the *more cooperating behaviours* (β = -0.34; p = 0.002) *sub-domains* tended to decrease with ASD children’s age. No significant effects of Age were reported in the TD group neither on *mean positive* (‘ < 6 y.o.’[N = 28]: 1.02 ± 1.04; ‘ ≥ 6 y.o.’[N = 12]: 0.83 ± 0.75; U = 162, p = 0.86) nor on *mean negative* mood changes (‘ < 6 y.o.’[N = 28]: 1.47 ± 0.64; ‘ ≥ 6 y.o.’[N = 13]: 1.53 ± 0.74; U = 170, p = 0.75).

Finally, concerning the Cognitive Level predictor (‘intellectual-disability’ Vs. ‘no-intellectual-disability’), in the ASD group no significant effects were found neither on *mean positive* (‘intellectual-disability’[N = 54]: 1.42 ± 0.89; ‘no-intellectual-disability’[N = 32]: 1.14 ± 0.80; U = 699, p = 0.14) nor on *mean negative* (‘intellectual-disability’[N = 54]: 1.71 ± 0.89; ‘no-intellectual-disability’[N = 32]: 1.88 ± 0.84; U = 770, p = 0.40) mood changes.

### Changes in core autistic features

Core autistic features reflect *domains* and *sub-domains* ascribable to the autistic functioning. Analyses aimed to test if context-specific critical variables have an impact on condition-specific *(sub-)domains*.

We found Group (ASD Vs. TD) differences in reporting changes in the *Sensory Interests* (ASD: 25%[44/176]; TD: 3%[3/86]; LRT-χ^2^ = 17.31, p < 0.001) and *Repetitive Behaviours* (ASD: 29%[52/178]; TD: 3%[3/86]; LRT-χ^2^ = 24.93, p < 0.001) *domains.* Among the *types-of-change* more specifically reflecting core autistic characteristics, we found that ASD children were reported to show more changes than TD ones in the *food selectivity* (ASD: 68%[26/38]; TD: 22%[4/18]; LRT-χ^2^ = 11.19, p < 0.001), and in the *difficulties with transition* (ASD[N = 77]: 1.91 ± 1.18; TD[N = 25]: 0.72 ± 1.02; β = 0.84; p < 0.001) *sub-domains.*

**Figure 3:**
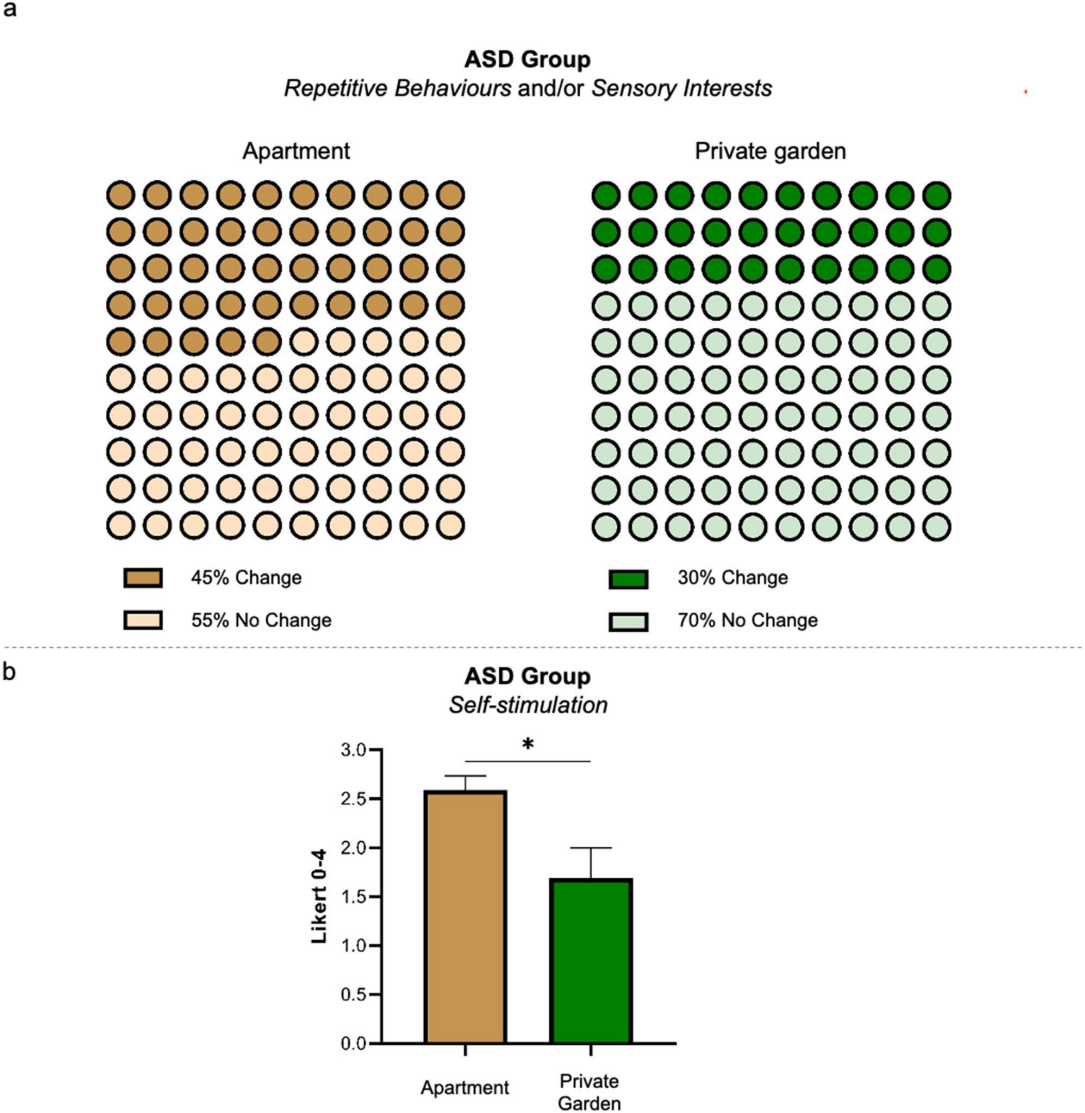
(**a**) Graphs illustrating the effects of House-Characteristics (apartment Vs. private garden) on *Repetitive Behaviours* and/or *Sensory Interests domains* within the ASD group (p = 0.036). (**b**) Graphs illustrating the effects of House-Characteristics (apartment Vs. private garden) on the *self-stimulation sub-domain* within the ASD group (p = 0.029). *Statistical significance, p < 0.05. Bars represent standard errors of the mean (SEM).

As expected, the number of TD children showing any changes in the autistic core *(sub-)domains* was basically negligible. Thus, we proposed a specific intra-group focus on the ASD sample. This choice was also justified by the fact that these *(sub-)domains* refer to aspects taken into account during clinical rehabilitative protocols, therefore children with ASD’s parents are familiar with them. In contrast, they may appear less transparent for neurotypical children’s parents. Although we clinically hypothesized a potential beneficial effect of accessing to private outdoor spaces on core autistic *domains*, the effect of House-Characteristics (apartment Vs. private garden) did not attain (*Repetitive Behaviours*, apartment: 32%[38/118]; private garden: 23%[14/60]; LRT-χ^2^ = 2.12, p = 0.15) or at-best approached the statistical significance level (*Sensory Interests*, apartment: 29%[34/116]; private garden: 17%[10/60]; LRT-χ^2^ = 3.35, p = 0.067). Driven by the clinical hypothesis that considers patterns of repetition and sensory processing peculiarities in ASD as potentially related to a common atypical functioning^30^, we set an additional variable testing the co-occurrence of changes in at-least-one *domain* (*Repetitive Behaviours and/or Sensory Interests*). This new variable confirmed that House-Characteristics predictor (apartment Vs. private garden) significantly modulate sensorial and repetitive patterns changes, as ASD children living in apartment were reported to show more changes in *Repetitive Behaviours* and/or *Sensory Interests* (apartment: 45%[53/118]; private garden: 30%[18/60]; LRT-χ^2^ = 4.41, p = 0.036) *domains* (Fig. 3a).

Among the *types-of-change* specifically reflecting core autistic features, the House-Characteristics predictor (apartment Vs private garden) similarly impacted on *self-stimulation* (apartment[N = 34]: 2.59 ± 0.86; private garden[N = 13]: 1.69 ± 1.11; β = 0.66; p = 0.029), denoting again that children without access to private garden were reported to show more changes (Fig. 3b). Notably, we also found a significant Age effect on *self-stimulation* (β = 0.36; p = 0.008), indicating that older children were reported to show more changes in this *sub-domain*.

## Discussion

Governments worldwide are trying to face this pandemic providing health policies that balance distinct aspects such as epidemiological situation, socioeconomic and biopolitics factors^10,11,31^. Although expression “unprecedented times” is becoming a sort of *cliché* in the COVID-19 era^32^, are we really transferring this idea and the burden of its implications in the mental health clinical settings? Are we really dealing with the lockdown effects on individuals with ASD taking into account the unique scenario of COVID-19 pandemic? *AutiStress* tries to tackle this point balancing peculiarities of the current situation, the body of knowledge on ASD (≈ *autistic functioning*), and notably the interaction of these aspects.

Our first result concerns the *Mood domain*, that refers to an unspecific and non-core autistic feature. As expected, the severe lockdown policies in Italy had a general negative impact both on children with ASD and neurotypicals controls. This generic and unsurprising result reflects the obvious burden of the pandemic situation, and it was a widely predictable effect of unpleasant (but undelayable) movement restrictions. Notably, we found that *mean negative* mood changes were higher than *mean positive* ones in each group (see also Tables S5, S6). This suggests that the effect was not condition-specific for the ASD group whereas it is shared also by our neurotypical pediatric sample, in agreement also with previous reports on clinical/non-clinical populations^4–6^. However, and importantly considering *AutiStress* aims, children with ASD reported more *positive* mood changes than TD children (Fig. 1c); notably, they have been reported to show more changes in the *more calm*, in the *more helpful*, and in the *more cooperating behaviours sub-domains*. Although the mainstream view tended to focus on the presumed amplified burden in ASD^25,27,28^, the possibility that our clinical sample may experience *also* positive changes during the COVID-19 pandemic cannot be considered totally unexpected. As outlined in the Introduction, children with ASD may have benefited from reduced stressful situations (e.g., public transports, mandatory school activities) and from new more manageable home-based routines. This may be particularly plausible in our ASD sample, considering that all children pursued readapted remote clinical intervention provided also during the more critical period. This may have guaranteed at least basic continuity of care, notably supporting parents in structuring and managing daily activities, and it is consistent with other recent reports describing beneficial effects of remote/telehealth programs in ASD^33–35^. Our data also suggest an interesting double dissociation in modulating *positive* mood changes between the two groups (Fig. 2). Age seems to explain *positive* changes in the ASD group, with younger children reported to show more changes in the *more calm*, in the *more helpful*, and in the *more cooperating behaviours sub-domains*. These findings may partially reflect a sort of “protective” role of age, being younger children naturally less aware of the situation, and also the new home-based routines established by caregivers supported by our clinical staff (see also^36^). In contrast, Age does not seem to play a critical role in explaining *positive* changes in the TD group. Intriguingly, an additional dissociation emerged for the House-Characteristics predictor that significantly predict *positive* changes in the TD group. Children with access to private outdoor spaces were reported to show more changes in the *more calm* and in the *more cooperating behaviours sub-domains*. In contrast, House-Characteristics does not seem to play a role in explaining *positive* changes in the ASD group. Taken together, results on the *Mood domain* depict a scenario largely consistent with the previous literature, with obvious negative effects of the severe anti-contagion policies impacting both on ASD and TD children. However, the double dissociation on *positive* changes may assume strong theoretical and clinical significance taking into account the autistic functioning peculiarities, and notably the distinction between core Vs. non-core autistic features.

A very timely report faced the challenge of setting up remotely readapted protocols^37^. This report has the merit of pushing readers in considering the peculiar core of the autistic phenotype, and nicely fits with the *AutiStress* purpose of differentiating changes in core Vs. non-core autistic features*.* Thus, our second major finding concerns a condition-related impact of severe anti-contagion policies on specific core autistic features. We found that children with ASD reported changes more often than TD controls in the *Sensory Interests* and *Repetitive Behaviours domains*. In addition, children with ASD reported significantly more changes than controls in *food selectivity* and in *difficulties with transitions*, that represent two prototypical *sub-domains* related to the autistic functioning (i.e., inflexibility, rigidity). From the one side, it is not surprising that neurotypical children basically did not present any changes in these aspects, and likely these requests may have appeared less transparent for neurotypical children’s parents. However, percentages and rough proportion in the ASD sample clearly indicate relevant changes compared to pre-pandemic period in these condition-specific aspects. Intriguingly, a subtler and deeper analysis of our ASD sample suggests an additional thought-provoking dissociation with the neurotypical control group. Considering ASD children reporting changes in at-least-one of the autistic core *domains* (*Sensory Interests* and/or *Repetitive Behaviours*), a significant effect of House-Characteristics emerged. Moreover, House-Characteristics significantly impacts on a *sub-domain* distinctive of the autistic functioning such as *self-stimulation* (Fig. 3). Results on House-Characteristics in the ASD sample hint at refined and clinically insightful perspective-shift. Our suggestion is that a context-specific critical variable may have a relevant impact on condition-specific *(sub-)domains* (i.e., core autistic features for the ASD group), whereas it would impact on a more general and unspecific domain such as *Mood* in neurotypical controls. A provocative hypothesis is that *Mood domain* for TD children corresponds to core autistic *domains* for the ASD group, as it was the “core neurotypical domain”. Although this remains a rather speculative hypothesis, our results clearly indicate a different effect of context-specific protective factors, and implicitly call for a multi-layered, condition-specific and less simplistic analyses of complex phenomena. Having access to private outdoor places reasonably represents a critical variable during severe lockdown. Potential mental health and well-being benefits of time spent outside in natural environments have been largely hypothesized in the literature^38^, notably in coping with strict movement restrictions^39^. Countries such as Italy and Spain promoted very restrictive lockdown policies in which people basically were not allowed to leave their homes except for healthy (e.g., brief outside walks were permitted in the Lombardy region for people with severe mental health disorders or intellectual disabilities, provided that the caregiver presented a written certificate signed by the clinical mental health specialist, see^37^), emergency or essential job reasons, for buying food and medicines, or—in certain cases—walking the dog. Even in a scenario of national lockdown, other countries permitted at least limited access to nature or public outdoor spaces for recreational activities (e.g., France, UK). Thus, accessibility to private outdoor spaces (e.g., private garden) is likely more critical in areas, such as Italy or Spain, in which not even limited access to public park or greenspace was permitted. This hypothesis not only drove our choices in *AutiStress* of controlling house characteristics, but it also nicely fits with the findings recently reported in a large sample of 3403 individuals from Spain^39^. The authors tested the potential resilient effect of the residual contact with the nature, and results suggest that having access to private outdoor spaces and window views of natural features (as a proxy of indirect contact with nature, see^40^) can act as a protective factor against negative consequences of severe stay-at-home restrictions. Without underestimating or even neglecting the role of other critical moderators (e.g., socio-demographic factors, personal circumstances), accessibility to private outdoor places may effectively represent a relevant factor in this pandemic. Accordingly, also our results indicate that private garden availability plays a role in coping the strict movement restrictions policies. More interestingly for our aims, we showed that children with ASD and neurotypical controls seem to be impacted *differently* from this modulator factor, as if it acted primarily on each individual’s more “sensible” spheres. Here again, no doubt that House-Characteristics is not the only ingredient modulating individual reaction to the pandemic. For children with ASD a primary role was also played by familiar coping strategies, eventually supported by remote programs guaranteeing continuity of cares.

Finally, a methodological point deserves to be underlined. It would be virtually meaningless considering each *(sub-)domain *per se, without a within-subject pre-/post-pandemic comparison. Two distinct approaches may solve this concern. The first one consists in the comparison of results on standardized questionnaires used during periodic clinical follow-up (as proxy of pre-pandemic timepoint) with a new re-evaluation ideally administered just before the relaxing of restrictions (as proxy of post-lockdown timepoint)^41–43^. This strategy has the undeniable advantage of using standardized instruments, however these instruments are not context*-*specific and usually not even condition*-*specific. A second alternative approach consists in ad hoc questionnaires. *AutiStress* clearly accompanied the respondents to not consider *absolute rate* for each *(sub-)domain*, whereas we drove them in focusing on potential *changes* in relation to pre-pandemic period. Noteworthy, *AutiStress* is context*-*specific: it is tailored to the COVID-19 pandemic situation (e.g. effects of enforced stay-at-home orders), and this inspired us in using ad hoc predictor (e.g., House-Characteristics) that would not be so befitting in other emergency contexts such as wars or natural disasters areas. *AutiStress* is also condition-specific: it is based on a renovated clinical framework that considers sensory processing and behavioural rigidity as the key components of ASD (≈ *autistic functioning*) that, in turn, inspired us in differentiating core Vs. non-core autistic features. Finally, asking for *changes* we implicitly “controlled” for a sort of individual threshold, and this helps to explain the modest impact of ASD children’s cognitive level in our results. At first glance this result may appear weird, being clinically well-established that certain components are normally more pronounced in ASD children with lower cognitive abilities (e.g., *self-stimulation* for which—indeed—results indicate a non-significant trend). Thus, we speculate that the emphasis on reporting *changes* may explain the absence of any significant results of the cognitive level (*domains,* all ps > 0.22; *sub-domains*, all ps > 0.11).

## Conclusion

The health, social and economic cost of COVID-19 pandemic is impressive^4–9,12,24–28,33–37,41–44^, and by now no one can fully evaluate neither short- nor long-term effects in a reliable way. Although our work offers only a very specific and circumscribed view, we hope this may contribute to improve global attention also to the (a)typical mental health impact of COVID-19 pandemic.

We intended *AutiStress* both as a *case-specific instrument* and a *potentially replicable tool*, as long as specific (but finally minimal) adaptations are provided. *AutiStress* strengthens the idea that we would benefit from an approach that considers seriously the autistic functioning and its learning/thinking/acting peculiarities, since extraordinary situations may selectively impact on them. This does not imply that more classical works focusing on general mental health symptoms regardless of the specificities of the clinical population are less informative, in light for example of the clear benefit of having access to standardized instruments. We simply state that they are not enough, and we should promote convergent/combined approaches that consider both perspectives. Similarly, facing an “unprecedented” emergency scenario we need peculiar contextual constraints (e.g., access to outdoor space). How we act and *inter*act in the world is a puzzle even during *golden age* periods. Promoting the best possible equilibrium between *safe* and *effective* social dynamics during this pandemic time seems to be a matter of titanic importance both for neurotypical and autistic population. We are all main characters of this challenge.

## Supplementary Information

Supplementary Tables.

## Data Availability

Materials and data generated during the current study and supporting the findings of this article are available from the corresponding authors upon reasonable request.
