# Supplementary material for: The (a)typical burden of COVID-19 pandemic scenario in Autism Spectrum Disorder
Source: Sci Rep. 2021 Nov 22;11:22655. doi: 10.1038/s41598-021-01907-x (PMC8608876; doi:10.1038/s41598-021-01907-x)
Supplement: Supplementary file 1 — Supplementary Tables. [file 41598_2021_1907_MOESM1_ESM.pdf]

The (a)typical burden of COVID-19 pandemic scenario in Autism Spectrum Disorder

Fumagalli L., Nicoli M., Villa L., Riva V., Vicovaro M., Casartelli L.

SUPPLEMENTARY TABLES

TableS1. The *AutiStress* questionnaire was originally created in Italian and distributed using Google Forms to maximize user-friendliness. Here, in the interest of readers, we provide an ad hoc English translation of the original questionnaire that – for the sake of simplicity – has been slightly readapted in its form without compromising the original structure. Answer type (open-ended, dichotomic or Likert-scale from 0 to 4) is reported for each question.

AutiStress Questionnaire

SECTION 1: Socio-demographic questions

Mother's age *[open-ended]*
Mother's nationality *[open-ended]*
Father's age *[open-ended]*
Father's nationality *[open-ended]*
House characteristics:<br>- apartment with balcony<br>- apartment without balcony<br>- house with private garden<br>- terraced house
Could you access to private outdoor spaces? *[yes/no]*
Who lives with your child? *[open-ended]*
Is there anyone that does not live with your child but is spending time with him/her (e.g., grandparents, babysitter, etc.)? *[open-ended]*
Are you keeping in touch with the nursery school? *[Rate from 0 (never) to 4 (always)]*
Is the mother currently employed? *[yes/no]*<br>*If so:*<br>- She is working in her usual workspace<br>- She is working from home
Is the father currently employed? *[yes/no]*<br>*If so:*<br>- He is working in her usual workspace<br>- He is working from home

SECTION 2: Domains and sub-domains

Did your child show any change in ***Circadian Rhythm Sleep***? *[yes/no]*

If so:

- Did your child show ***difficulty falling asleep***?
  [Rate from 0 (never) to 4 (always)]
- Did your child show ***early awakenings***?
  [Rate from 0 (never) to 4 (always)]
- Did your child ***return to parents' bed***?
  [Rate from 0 (never) to 4 (always)]
- Did your child show ***nocturnal pavor or sonnambulism***?
  [Rate from 0 (never) to 4 (always)]

Did your child show any change in ***Eating Behaviour***? *[yes/no]*

If so:

- Did your child show ***food selectivity***?
  [Rate from 0 (never) to 4 (always)]
- Did your child ***food selectivity increase***?
  [Rate from 0 (never) to 4 (always)]
- Did your child ***eat more***?
  [Rate from 0 (never) to 4 (always)]
- Did your child ***eat less***?
  [Rate from 0 (never) to 4 (always)]
- Did your child ***need to be feeded***?
  [Rate from 0 (never) to 4 (always)]
- Did your child ***change meal hours***?
  [Rate from 0 (never) to 4 (always)]

Did your child show any change in ***Sensory Interests***? *[yes/no]*

If so:

- Interest in the sense of ***touch***: *[Choose and Rate from 0 (never) to 4 (always)]*
  - Increased
  - Decreased
  - Unchanged
- Interest in the sense of ***sight***: *[Choose and Rate from 0 (never) to 4 (always)]*
  - Increased
  - Decreased
  - Unchanged
- Interest in the sense of ***hearing***: *[Choose and Rate from 0 (never) to 4 (always)]*
  - Increased
  - Decreased
  - Unchanged
- Interest in the sense of ***smell***: *[Choose and Rate from 0 (never) to 4 (always)]*
  - Increased
  - Decreased
  - Unchanged
- Interest in the sense of ***taste***: *[Choose and Rate from 0 (never) to 4 (always)]*
  - Increased
  - Decreased
  - Unchanged

Did your child show any change in ***Play***? *[yes/no]*
If so:<br>- Did your child ***increase the number of his/her interests***?<br>*[Rate from 0 (never) to 4 (always)]*<br>- Did your child ***reduce the number of his/her interests***?<br>*[Rate from 0 (never) to 4 (always)]*<br>- Did your child ***hyper-focus on some activity***?<br>*[Rate from 0 (never) to 4 (always)]*<br>- Did your child's ***inattention increase***?<br>*[Rate from 0 (never) to 4 (always)]*<br>- Did your child show ***difficulties with transitions***?<br>*[Rate from 0 (never) to 4 (always)]*
Did your child show any change in ***Repetitive Behaviours***? *[yes/no]*
If so:<br>- ***Stereotypies***: *[Choose and Rate from 0 (never) to 4 (always)]*<br>▪ Increased<br>▪ Modified<br>- ***Self-stimulation***: *[Choose and Rate from 0 (never) to 4 (always)]*<br>▪ Increased<br>▪ Modified
Did your child show any change in ***Mood***? *[yes/no]*
If so:<br>- Was your child ***more irritable***? **(-)**<br>*[Rate from 0 (never) to 4 (always)]*<br>- Was your child ***more whiny***? **(-)**<br>*[Rate from 0 (never) to 4 (always)]*<br>- Did your child ***cry for no reason***? **(-)**<br>*[Rate from 0 (never) to 4 (always)]*<br>- Did your child show ***intolerance to frustration***? **(-)**<br>*[Rate from 0 (never) to 4 (always)]*<br>- Did your child show ***more oppositional behaviours***? **(-)**<br>*[Rate from 0 (never) to 4 (always)]*<br>- Was your child ***more provocative***? **(-)**<br>*[Rate from 0 (never) to 4 (always)]*<br>- Was your child ***more calm***? **(+)**<br>*[Rate from 0 (never) to 4 (always)]*<br>- Did your child show ***more cooperating behaviours***? **(+)**<br>*[Rate from 0 (never) to 4 (always)]*<br>- Was your child ***more helpful***? **(+)**<br>*[Rate from 0 (never) to 4 (always)]*
Did your child show any change in ***Bowel and Bladder control***? *[yes/no]*
If so:<br>- Did your child show nocturnal/diurnal ***loss/gain of bowel control***?<br>*[Rate from 0 (never) to 4 (always)]*<br>- Did your child show nocturnal/diurnal ***loss/gain of bladder control***?<br>*[Rate from 0 (never) to 4 (always)]*

Answer type = *[open-ended]*; *[yes-no]*; *[rate from 0 (never) to 4 (always)]*
(-) = *negative* mood change; (+) = *positive* mood change

TableS2. Significant Group effects (ASD Vs. TD) in non-core *(sub-)domains*

**MAIN EFFECTS of GROUP**				
DOMAINS				
DOMAIN	**% ASD**	**% TD**	**p-value LRT χ^2**	**p-value K-S test**
Play	46% (82/178)	30% (26/86)	p=.033	--
Bower and Bladder Control	12% (20/172)	3% (3/86)	p=.040	--
Circadian Rhythm Sleep	49% (88/178)	35% (30/86)	p=.015	--
SUB-DOMAINS				
SUB-DOMAIN	**% ASD**	**% TD**	**p-value LRT χ^2**	**p-value K-S test**
"Early awakenings" *(domain: Cyrcadian Rhythm Sleep)*	51% (30/59)	26% (5/19)	p=.046	p<.001
"Need to be feeded" *(domain: Eating Behavior)*	63% (24/38)	28% (5/18)	p=.009	p=.025
"He/she eats less" *(domain: Eating Behavior)*	49% (19/39)	22% (4/18)	p=.042	p<.001
"Inattention" *(domain: Play)*	83% (63/76)	64% (16/25)	p=.019	p<.001

LRT = likelihood ratio test; K-S=Kolmogorov-Smirnov normality test; for the *sub-domains*, the results refer only to the participants who reported a change in the corresponding domain.

TableS3. Significant House-Characteristics effects (apartment Vs. private garden) in *(sub-)domains* within the TD group

MAIN EFFECTS of HOUSE-CHARACTERISTICS				
SUB-DOMAINS				
SUB-DOMAIN	**% Apartment**	**% Private Garden**	**p-value LRT χ^2**	**p-value K-S test**
"Decrease of interests" *(domain: Play)*	83% (10/12)	42% (5/12)	p=.033	p=.040

LRT = likelihood ratio test; K-S=Kolmogorov-Smirnov normality test; the result refers only to the participants who reported a change in the corresponding domain.

TableS4. Significant Age effects in non-core *(sub-)domains* within the ASD group

MAIN EFFECTS of AGE			
DOMAINS			
DOMAIN	**Exp(b)**	**p-value LRT χ^2**	**p-value K-S test**
Bower and bladder control	0.751	p=.040	--
SUB-DOMAINS			
SUB-DOMAIN	**Exp(b)**	**p-value LRT χ^2**	**p-value K-S test**
"He/she eats more" *(domain: Eating Behavior)*	0.578	p=.016	p=.035

LRT = likelihood ratio test; K-S=Kolmogorov-Smirnov normality test; Exp(b)=exponentiation of b coefficient (odd ratio); for the *sub-domains*, the result refers only to the participants who reported a change in the corresponding domain.

TableS5. Comparisons between each *positive* and each *negative* mood *sub-domain* within the ASD group. Because neither *positive* nor *negative types-of-change* were normally distributed, we used Wilcoxon Paired Tests to compare *positive* Vs. *negative* differences in each *positive* and each *negative sub-domain*. Significance threshold was adjusted at p=.003, according to Bonferroni correction for multiple comparisons (p=.05/18=.003). All significant results show that *positive* mood *types-of-change* are reported less frequently than *negative* ones.

ASD GROUP				
***Positive* mood sub-domain**	**Mean ± SD (N)**	***Negative* mood sub-domain**	**Mean ± SD (N)**	**p-value**
More calm	1.30 ± 1.03 (N=82)	*More irritable*	1.90 ± 1.05 (N=82)	p=.002*
	1.29 ± 1.02 (N=80)	*More whiny*	1.91 ± 1.09 (N=80)	p<.001*
	1.28 ± 1.02 (N=81)	*Cry for no reason*	1.14 ± 1.13 (N=81)	p=.247
	1.30 ± 1.03 (N=82)	*Intolerance to frustration*	2.24 ± 1.19 (N=82)	p<.001*
	1.31 ± 1.03 (N=81)	*Oppositional behaviour*	1.88 ± 1.17 (N=81)	p=.002*
	1.30 ± 1.03 (N=82)	*More provocative*	1.67 ± 1.29 (N=82)	p=.101
More cooperating behaviours	1.24 ± 0.91 (N=84)	*More irritable*	1.89 ± 1.04 (N=84)	p<.001*
	1.23 ± 0.92 (N=82)	*More whiny*	1.91 ± 1.09 (N=82)	p<.001*
	1.23 ± 0.92 (N=83)	*Cry for no reason*	1.14 ± 1.12 (N=83)	p=.440
	1.24 ± 0.91 (N=84)	*Intolerance to frustration*	2.24 ± 1.18 (N=84)	p<.001*
	1.24 ± 0.92 (N=83)	*Oppositional behaviour*	1.86 ± 1.17 (N=83)	p=.001*
	1.25 ± 0.91 (N=83)	*More provocative*	1.69 ± 1.29 (N=83)	p=.052
More helpful	1.37 ± 0.92 (N=83)	*More irritable*	1.92 ± 1.03 (N=83)	p=.006
	1.36 ± 0.93 (N=81)	*More whiny*	1.91 ± 1.10 (N=81)	p=.003*
	1.37 ± 0.92 (N=82)	*Cry for no reason*	1.16 ± 1.12 (N=82)	p=.178
	1.37 ± 0.92 (N=83)	*Intolerance to frustration*	2.25 ± 1.18 (N=83)	p<.001*
	1.37 ± 0.92 (N=82)	*Oppositional behaviour*	1.88 ± 1.16 (N=82)	p=.006
	1.39 ± 0.91 (N=82)	*More provocative*	1.68 ± 1.30 (N=82)	p=.122

* = results that survived the correction for multiple comparisons.

TableS6. Comparisons between each *positive* and each *negative* mood *sub-domain* within the TD group. Because neither *positive* nor *negative types-of-change* were normally distributed, we used Wilcoxon Paired Tests to compare *positive* Vs. *negative* differences in each *positive* and each *negative sub-domain*. Significance threshold was adjusted at p=.003, according to Bonferroni correction for multiple comparisons (p=.05/18=.003). All significant results show that *positive* mood *types-of-change* are reported less frequently than *negative* ones.

**TD GROUP**				
Positive* mood *sub-domain	**Mean ± SD (N)**	***Negative* mood *sub-domain***	**Mean ± SD (N)**	**p-value**
More calm	0.69 ± 0.86 (N=39)	*More irritable*	1.80 ± 0.77 (N=39)	p<.001*
	0.69 ± 0.86 (N=39)	*More whiny*	1.90 ± 0.82 (N=39)	p<.001*
	0.69 ± 0.86 (N=39)	*Cry for no reason*	0.69 ± 0.92 (N=39)	p=.872
	0.69 ± 0.86 (N=39)	*Intolerance to frustration*	1.77 ± 1.16 (N=39)	p<.001*
	0.69 ± 0.86 (N=39)	*Oppositional behaviour*	1.64 ± 0.90 (N=39)	p<.001*
	0.69 ± 0.86 (N=39)	*More provocative*	1.31 ± 1.06 (N=39)	p=.014
More cooperating behaviours	1.08 ± 1.05 (N=40)	*More irritable*	1.78 ± 0.77 (N=40)	p=.003*
	1.08 ± 1.05 (N=40)	*More whiny*	1.88 ± 0.82 (N=40)	p=.001*
	1.08 ± 1.05 (N=40)	*Cry for no reason*	0.68 ± 0.92 (N=40)	p=.075
	1.08 ± 1.05 (N=40)	*Intolerance to frustration*	1.75 ± 1.15 (N=40)	p=.004
	1.08 ± 1.05 (N=40)	*Oppositional behaviour*	1.60 ± 0.93 (N=40)	p=.033
	1.08 ± 1.05 (N=40)	*More provocative*	1.33 ± 1.05 (N=40)	p=.288
More helpful	1.08 ± 1.16 (N=39)	*More irritable*	1.80 ± 0.77 (N=39)	p=.007
	1.08 ± 1.16 (N=39)	*More whiny*	1.90 ± 0.82 (N=39)	p<.001*
	1.08 ± 1.16 (N=39)	*Cry for no reason*	0.69 ± 0.92 (N=39)	p=.122
	1.08 ± 1.16 (N=39)	*Intolerance to frustration*	1.77 ± 1.16 (N=39)	p=.006
	1.08 ± 1.16 (N=39)	*Oppositional behaviour*	1.64 ± 0.90 (N=39)	p=.021
	1.08 ± 1.16 (N=39)	*More provocative*	1.31 ± 1.06 (N=39)	p=.476

* = results that survived the correction for multiple comparisons.
